# Estradiol-17β-Induced Changes in the Porcine Endometrial Transcriptome In Vivo

**DOI:** 10.3390/ijms21030890

**Published:** 2020-01-30

**Authors:** Piotr Kaczynski, Stefan Bauersachs, Monika Baryla, Ewelina Goryszewska, Jolanta Muszak, Waldemar J. Grzegorzewski, Agnieszka Waclawik

**Affiliations:** 1 Institute of Animal Reproduction and Food Research, Polish Academy of Sciences, Tuwima 10, 10-748 Olsztyn, Poland; p.kaczynski@pan.olsztyn.pl (P.K.); m.baryla@pan.olsztyn.pl (M.B.); e.goryszewska@pan.olsztyn.pl (E.G.); 2 Vetsuisse Faculty, Clinic of Reproductive Medicine, Genetics and Functional Genomics, University of Zurich, Eschikon 27 EHB 23.1, 8315 Lindau, Switzerland; stefan.bauersachs@uzh.ch; 3 District Veterinary Inspectorate in Nidzica, Limanowskiego 1, 13-100 Nidzica, Poland; jolantamuszak@wp.pl; 4 Department of Animal Physiology and Reproduction, Institute of Biology and Biotechnology, College of Natural Sciences, University of Rzeszow, Pigonia 1, 35-310 Rzeszów, Poland; wgrzegorzewski@ur.edu.pl

**Keywords:** estradiol-17β, early pregnancy, endometrium, transcriptome profiling, pig, *Sus scrofa*

## Abstract

Estradiol-17β (E2) is a key hormone regulating reproductive functions in females. In pigs, E2, as the main conceptus signal, initiates processes resulting in prolonged corpus luteum function, embryo development, and implantation. During early pregnancy the endometrium undergoes morphological and physiological transitions that are tightly related to transcriptome changes. Recently, however, the importance of E2 as a primary conceptus signal in the pig has been questionable. Thus, the aim of the present study was to determine the effects of E2 on the porcine endometrial transcriptome in vivo and to compare these effects with transcriptome profiles on day 12 of pregnancy. Microarray analysis revealed differentially expressed genes (DEGs) in response to E2 with overrepresented functional terms related to secretive functions, extracellular vesicles, cell adhesion, proliferation and differentiation, tissue rearrangements, immune response, lipid metabolism, and many others. Numerous common DEGs and processes for the endometrium on day 12 of pregnancy and E2-treated endometrium were identified. In summary, the present study is the first evidence for the effect of E2 on transcriptome profiles in porcine endometrium in vivo in the period corresponding to the maternal recognition of pregnancy. The presented results provide a valuable resource for further targeted studies considering genes and pathways regulated by conceptus-derived estrogens and their role in pregnancy establishment.

## 1. Introduction

Local autocrine-paracrine signaling at the embryo-maternal interface is crucial for the successful establishment of pregnancy. Reduced fertility is a serious problem in animal and human reproduction. Pregnancy losses significantly decrease the profitability in animal production. The reasons for unsuccessful implantation are associated with the embryo e.g., insufficient conceptus signaling [1], genetic disorders, and impaired endometrial receptivity (reviewed in [2]). In most mammals, estradiol-17β (E2) serves as a pivotal molecule regulating reproductive functions of the female reproductive tract. In humans and rodents, estrogens secreted by ovarian follicles prior to ovulation are required for regeneration and growth of the endometrium and prime the tissue for progesterone (P4) (reviewed in [3]). Progesterone, called a “pregnancy hormone”, in turn regulates endometrial receptiveness, enabling conceptus implantation and early pregnancy development (reviewed in [4]). During early pregnancy in the pig, porcine conceptuses (embryos with associated membranes) secrete E2, which is regarded as the pregnancy recognition signal required for prolonged progesterone synthesis and secretion by corpora lutea (CLs). Porcine conceptuses secrete estrogens in a biphasic manner—the first increase of conceptus-derived estrogens occurs on days 11–13 after fertilization and the second is between days 15 and 25–30 after fertilization (reviewed in [2]). However, the levels of conceptus-derived estrogens in the uterine lumen may also vary depending on the number of conceptuses [1]. The period of elevated E2 synthesis and secretion by porcine conceptuses between days 11–13 of pregnancy is a process defined as the maternal recognition of pregnancy [5]. This is the critical period for establishment and development of pregnancy as the highest mortality rate of embryos in animals including pigs is observed during the peri-implantation period [6]. Embryonic estrogen synthesis and secretion not only prolongs the CL lifespan but also enhances P4-induced endometrial receptivity for implantation. Following the decrease of the progesterone receptor expression in the endometrial luminal and glandular epithelia, the expression of the estrogen receptor (ESR1) is up-regulated in these structures, which in turn is important for the cell-specific responses to conceptus estrogens released on day 12 of pregnancy [7]. Estrogen is involved in stimulation of uterine secretory activity [8], increased blood flow [9], endometrial edema [10], and the regulation of endometrial expression of many factors (reviewed in [4]). Recent studies revealed that porcine conceptuses with an aromatase knock out (*CYP19A1*^−/−^) were capable of maintaining the corpora lutea and survived until day 27–30 of pregnancy [11]. Therefore, the role of E2 as a primary conceptus signal in the pig has recently been questionable.

Understanding the mechanisms of pregnancy development in animals, including pigs, requires establishing the hierarchy and timing of molecular relationships in the maternal–conceptus crosstalk. Recent studies on endometrial transcriptome profiling revealed that differentially expressed genes (DEGs) enriched a number of processes, including regulation of immune response, tissue rearrangements, cellular processes (adhesion, proliferation, and differentiation), and many others [12,13,14]. However, as porcine embryos secrete diverse factors, research on the endometrial transcriptome during pregnancy does not elucidate the particular role of the main embryonic signal (E2) in these processes.

Considering the above facts, we hypothesize that E2, acting as the main embryonic signal, induces changes in the porcine endometrial transcriptome and triggers the processes involved in embryo–maternal interactions during early pregnancy. In studies on the mechanisms of maternal recognition of pregnancy in pigs, usually in vivo models of pseudopregnancy are used, in which pharmacological concentrations of 5 mg of estradiol benzoate is administered systemically on days 11–15 of the estrous cycle [8,15,16,17]. Regarding pharmacological doses of E2 and the systemic route of administration of the hormone, conclusions from studies in which in vivo models of pseudopregnancy should be considered with careful attention.

Thus, concerning a significant role of E2 in endometrial function in most mammals and the divergent information about the importance of E2 signaling in the establishment and development of pregnancy in the pig, the aim of the present study was to determine the effect of E2 on porcine endometrial transcriptome using a novel in vivo model, in which E2 in concentrations similar to the physiological levels secreted by embryos on day 11–12 of pregnancy was administered directly into the uterine lumen. This approach allowed us to examine both direct and indirect local effects of E2 on differential gene expression in porcine endometrium. Moreover, we aimed to compare the changes of endometrial gene expression on day 12 of pregnancy to those induced by estrogen administration. This allows us to determine the processes activated or inhibited on day 12 of pregnancy related with estrogen signaling.

## 2. Results

### 2.1. Endometrial Transcriptome Profiling

Changes in the endometrial transcriptome profile towards estradiol-17β treatment in vivo and on day 12 of pregnancy compared to day 12 of the estrous cycle were determined using the porcine Agilent microarray assay. Differential gene expression between the analyzed groups (833 ng of E2/infusion vs. control, 33.3 μg of E2/infusion vs. control; day 12 of pregnancy vs. day 12 of the estrous cycle) was evaluated separately for each experiment. The numbers of detected probes passing the filters were as follows: 39,253 (833 ng of E2/infusion), 38,935 (33.3 μg of E2/infusion), and 37,426 (day 12 of pregnancy vs. day 12 of the estrous cycle).

**Figure 1:**
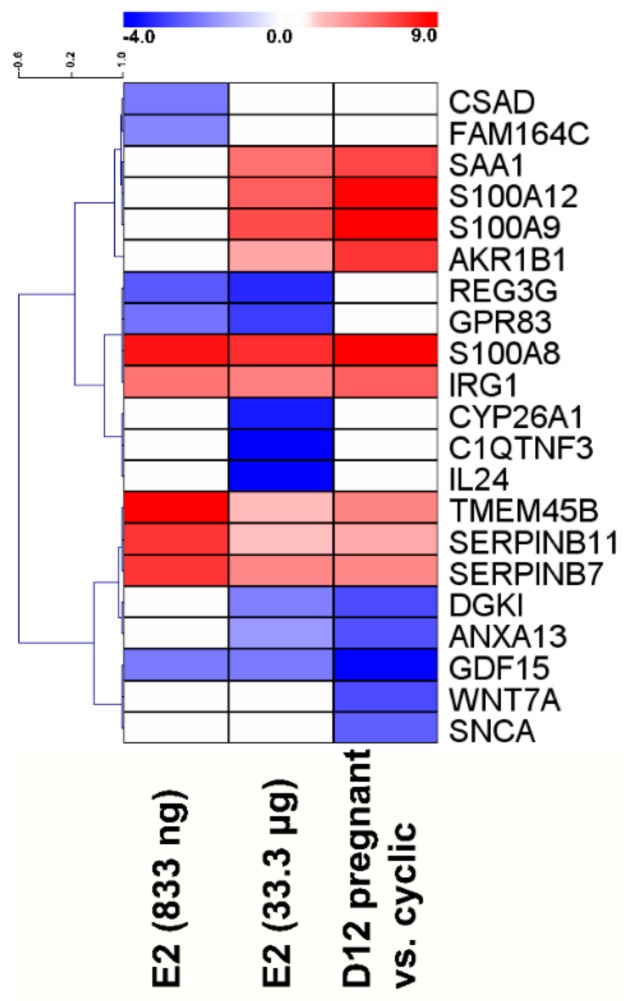
Heatmap of the log2 fold changes (estradiol-17β (E2)-treated endometrium vs. placebo-treated endometrium; day 12 pregnant-cyclic) of the top 10 differentially expressed genes of each gene expression comparison. The scale is from log2 fold change -4 (blue, down-regulated in E2-treated/pregnant samples) to 9 (red, up-regulated in E2-treated/pregnant samples). Each row represents one gene, and each column represents a comparison (E2-treated vs placebo or pregnant vs. cyclic).

Pairwise distance analysis based on all detectable probes revealed that endometrial samples collected from E2- and placebo-treated pigs represented mainly treatment grouping with a slight bias observed in the heatmap (Supplementary Figure S1A,B). Samples collected from gilts on day 12 of the pregnancy and day 12 of the estrous cycle were grouped by reproductive status (pregnant–cyclic; Supplementary Figure S1C). Using the LIMMA package, 434 (833 ng of E2/infusion), 3176 (33.3 μg of E2/infusion), and 1167 (day 12 of pregnancy vs. day 12 of the estrous cycle), probes were found as differentially regulated (fold change </>1.5; *p* < 0.05; false discovery rate (FDR) = 5%). Collapsing the results from the probe level to the gene level revealed 251 (833 ng of E2/infusion), 1957 (33.3 μg of E2/infusion), and 727 (day 12 of pregnancy vs. day 12 of the estrous cycle) differentially expressed genes (DEGs).The results of statistical analysis of microarray data are summarized in Supplementary Tables S1–S3. In endometrial samples collected from horns receiving 833 ng of E2/infusion, the five DEGs showing the highest upregulation in the pairwise comparison “E2-treated horns” vs. “placebo-treated horns” were *S100A8* (18.7-fold), *SERPINB7* (18.1-fold), *SERPINB11* (7-fold), *IRG1* (5.4-fold), and *TMEM45B* (5.3-fold), whereas the five most down-regulated genes were *REG3G* (6.1-fold), *GPR83* (4.6-fold), *GDF15* (4.3-fold), *CSAD* (4.3-fold), and *FAM164C* (3.7-fold; Figure 1).

In endometrial samples collected from horns receiving 33.3 μg of E2/infusion, the five most up-regulated genes were *S100A8* (150.2-fold), *S100A9* (78.6-fold), *S100A12* (50.7-fold), *SAA1* (33.4-fold), and *IRG1* (23.7-fold). The five most down-regulated genes were *IL24* (26.8-fold), *C1QTNF3* (14.8-fold), *CYP26A1* (11-fold), *REG3G* (9.8-fold), and *GPR83* (8.2-fold; Figure 1). The five DEGs showing the highest upregulation in the pairwise comparison “12 day of pregnancy” vs. “12 day of the estrous cycle” were *S100A8* (1221-fold), *S100A9* (907-fold), *S100A12* (368-fold), *AKR1B1* (127-fold), and *SAA1* (88-fold), whereas the five most down-regulated genes (suppressed on day 12 of pregnancy) were *GDF15* (14-fold), *WNT7A* (seven-fold), *DGKI* (seven-fold), *ANXA13* (six-fold), and *SNCA* (six-fold; Figure 1).

The Pearson correlation performed for DEGs detected in endometrial samples from in vivo experiments (833 ng of E2/infusion and 33.3 μg of E2/infusion) revealed treatment grouping of the analyzed samples with two main distinct clusters of genes with uniform distribution of expression signals (Supplementary Figures S2 and S3). In endometrial samples collected on day 12 of pregnancy and day 12 of the estrous cycle, the Pearson correlation revealed reproductive status grouping (pregnant–cyclic) of analyzed samples with two main distinct clusters of genes with uniform distribution of expression signals (Supplementary Figure S4).

### 2.2. Functional Annotation of Microarray Data

Functional terms enriched for up-regulated and down-regulated DEGs in the analyzed groups were identified by using the Database for Annotation, Visualization and Integrated Discovery (DAVID). For DEGs up-regulated in endometrial samples collected from gilts treated with E2 (833 ng E2/infusion), DAVID identified 36 annotation clusters (enrichment score 3.8–1.3) and for down-regulated genes 15 annotation clusters (enrichment score 1.9–1.3) were identified. For DEGs up-regulated in the group treated with 33.3 μg of E2 per infusion, DAVID identified 255 annotation clusters (enrichment score 30.1–1.3), whereas for down-regulated genes, 60 annotation clusters (enrichment score 27.6–1.3) were identified.

Functional annotation clustering performed for E2-up-regulated DEGs (833 ng/infusion) revealed terms related to glucose metabolic process, calcium ion transport, protein phosphorylation, positive regulation of apoptosis and cell growth, epithelial differentiation, cell adhesion and migration, response to hypoxia, placenta development, lipid transport, and regulation of blood circulation. The terms enriched for down-regulated genes included lipid and steroid metabolism, protein ubiquitination, calcium transport, and epithelial tube morphogenesis.

Genes up-regulated by E2 (33.3 μg) revealed enriched terms such as extracellular exosomes and vesicles, focal adhesion, cell junctions, regulation of the inflammatory response, cell migration, proliferation, apoptosis, the mitogen-activated protein kinase (MAPK) cascade, angiogenesis, cytokine production, leukocyte migration, NF-kappaB signaling, lipid metabolism, gland development, and the response to hypoxia. Functional annotation clustering of genes down-regulated by E2 (33.3 μg) revealed overrepresented functional terms related to negative chemotaxis, cell differentiation, cytoskeletal binding, histone acetylation, and import into the cell (Table 1). The complete results from the DAVID analysis performed for E2-affected DEGs are summarized in Supplementary Tables S4 and S5.

For DEGs up-regulated on day 12 of pregnancy, DAVID identified 98 annotation clusters (enrichment score 11.3–1.3) whereas for DEGs down-regulated on day 12 of pregnancy 20 annotation clusters (enrichment score 3.5–1.3) were identified. The most interesting functional terms of overrepresented annotation clusters enriched for pregnancy-associated up-regulated genes were related to vascular development, cell migration, extracellular vesicles, focal adhesion, and immune response activation (Table 1). For pregnancy-associated down-regulated genes, the most interesting enriched terms were related to lipid metabolism, fatty acid biosynthesis, regulation of cell morphogenesis and differentiation, and the response to hormone and transport (Table 1). The complete results from the functional annotation clustering performed for pregnancy-associated DEGs are summarized in Supplementary Table S6.

### 2.3. Comparison of Estradiol-17β Effect with Pregnancy-Induced Transcriptome Changes

**Figure 2:**
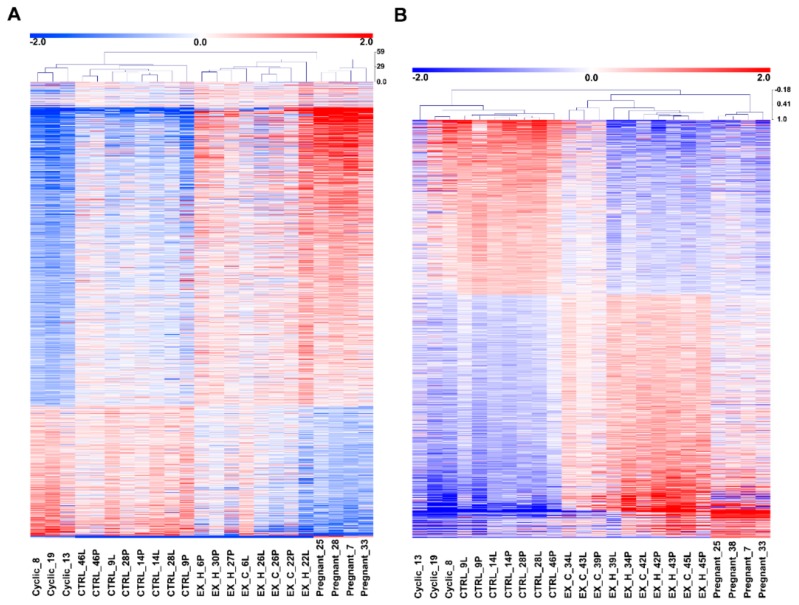
Comparison of transcriptome changes detected in endometrial samples collected from gilts on day 12 of the estrous cycle and pregnancy with the effect of intrauterine estradiol-17β administration (**A**: 833 ng of E2/infusion; **B**: 33.3 μg of E2/infusion). Data are shown as a heatmap of hierarchical clustering (Pearson correlation) of the normalized values of the intensity of the expression signals of probes detected as significantly changed (log fold change > 0.585; p nominal < 0.05 false discovery rate (FDR) = 5%) in analyzed groups. Cyclic-endometrial samples collected on day 12 of the estrous cycle, pregnant-endometrial samples collected on day 12 of pregnancy, control-endometrial samples collected from gilts assigned to control groups (infusions of placebo to both uterine horns), EX_H-endometrial samples collected from gilts assigned to the experimental group (uterine horns receiving E2 infusions), EX_C-endometrial samples collected from gilts assigned to experimental group (uterine horns receiving placebo infusion).

Pearson correlation of mean-centered values of expression signals of pregnancy-associated and E2-affected DEGs revealed pregnancy status (pregnant–non-pregnant) and treatment (E2, placebo) grouping (Figure 2A,B). Interestingly, endometrial samples collected from uterine horns receiving estradiol-17β infusion (833 ng and 33.3 μg/infusion) were grouped closer to the endometrial samples collected from gilts on day 12 of pregnancy, whereas the endometrial samples collected from uterine horns receiving placebo infusions were grouped closer to endometria from gilts on day 12 of the estrous cycle (Figure 2A,B).

**Figure 3:**
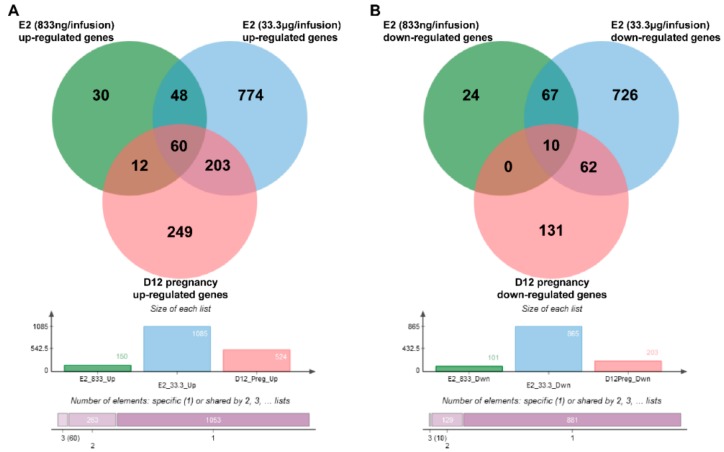
Venn diagrams of common, and group-specific up- (**A**) and down-regulated (**B**) genes. D12 pregnancy–endometrial samples collected on day 12 of pregnancy, E2 (833 ng/infusion) endometrial samples collected from uterine horns receiving 833 ng of E2/infusion, E2 (33.3 μg/infusion)—endometrial samples collected from uterine horns receiving 33.3 μg of E2/infusion. Complete lists of common and group-specific DEGs are presented in Supplementary Table S7.

Lists containing up- and down-regulated genes specific for each analyzed group were identified using jVenn software. Comparison of up-regulated genes identified in E2-treated endometria with genes that were found to be up-regulated in day 12 of pregnancy endometrial samples revealed 72 (833 ng of E2/infusion) and 263 (33.3 μg of E2/infusion) common genes. Sixty genes were found to be up-regulated in all analyzed groups. Comparison of down-regulated genes identified in E2-treated endometria with genes that were found to be down-regulated in day 12 of pregnancy endometrial samples revealed 10 (833 ng of E2/infusion) and 72 (33.3 μg of E2/infusion) common genes when compared to day 12 of pregnancy-down regulated genes. Ten genes were found to be down-regulated in all analyzed groups. One hundred and eight out of 150 (72%) up-regulated genes identified in endometrial samples treated with a lower dose of E2 (833 ng/infusion) were also up-regulated by a higher dose of E2 (33.3 μg/infusion). Seventy-seven out of 101 (76%) down-regulated genes were found to be common for E2 (833 ng/infusion)- and E2 (33.3 μg/infusion)-treated endometrial samples. Venn diagrams of common and group-specific DEGs are shown in Figure 3, and the complete results of these comparisons are summarized in Supplementary Table S7.

**Figure 4:**
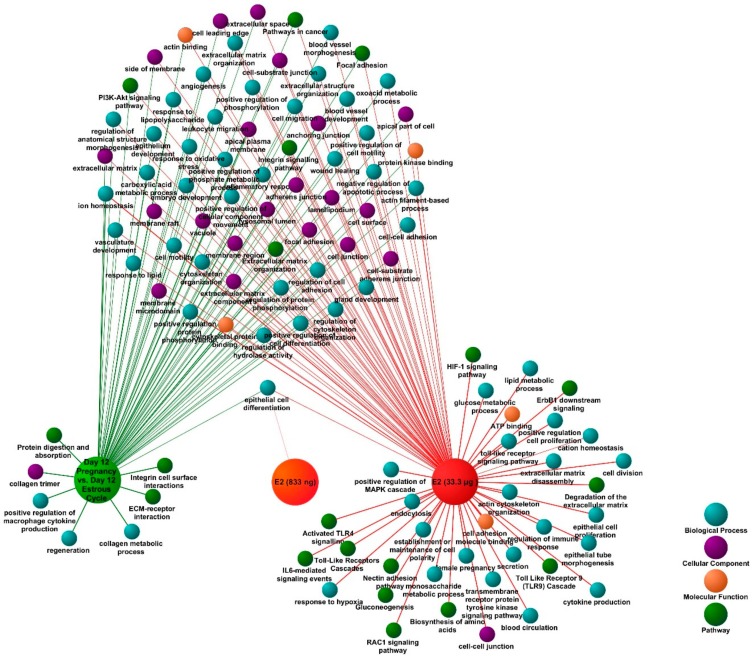
A functional map showing shared and group-specific functional annotation terms generated in multi-cluster gene functional enrichment analysis for differentially expressed genes (DEGs) identified in porcine endometrial samples collected on day 12 of pregnancy and treated with E2 (833 ng or 33.3 μg/infusion). Analysis was performed in ToppCluster software, the functional map was edited and adjusted using Cytoscape. The redundant and noninformative terms were removed. The complete results are presented in a tabular format in Supplementary Table S8.

A multiple gene list feature enrichment analyzer (Topp Cluster) was used to determine functional terms specifically related to DEGs identified for each analyzed group: E2 (833 ng/infusion; 33.3 μg/infusion), day 12 of pregnancy and the estrous cycle. Results of this analysis showed that the majority of identified enriched terms were in common for pregnancy-associated and E2-affected DEGs (Figure 4). These were related to extracellular matrix organization, angiogenesis, integrin signaling, epithelium development, embryo development, PI3K-AKT signaling, and leukocyte migration. However, several terms were found to be related only with day 12 of pregnancy-associated or E2-affected DEGs. The most interesting terms enriched by day 12 of pregnancy-associated DEGs were related with extracellular matrix (ECM)-receptor interaction, regeneration, protein digestion, and absorption, whereas terms enriched by E2 treatment were associated with activation of Toll like receptor 4 (TLR4) signaling, establishment or maintenance of cell polarity, epithelial cell proliferation, and the hypoxia-inducible factor 1 (HIF1) signaling pathway. The most interesting enriched terms from clusters identified for analyzed groups were visualized as a network (Figure 4). The complete results are provided in Supplementary Table S8.

**Figure 5:**
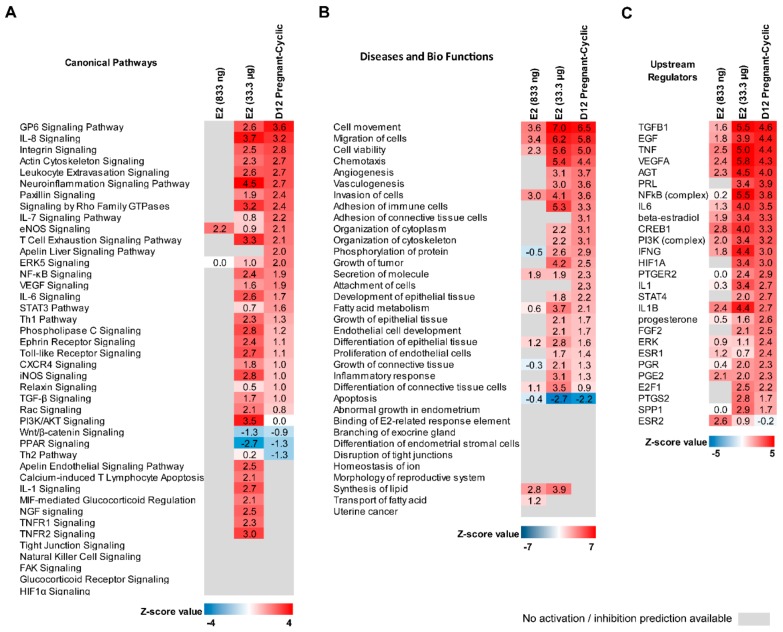
Comparison analysis of canonical pathways (**A**), diseases and bio functions (**B**) and upstream regulators enriched by DEGs identified in estradiol-17β-treated (833 ng and 33.3 μg/infusion) and day 12 of pregnancy endometrial samples. A Z-score value higher than 2 means that the enriched pathway (**A**) or enriched function (**B**) is significantly activated or stimulated, or the upstream regulator stimulates the set of identified DEGs (**C**). A Z-score value lower than 2 means that the enriched pathway (**A**) or enriched function (**B**) is significantly inhibited or suppressed, or the upstream regulator suppresses the set of identified DEGs (**C**). Grey color indicates there is no data allowing for activation/suppression or stimulation/inhibition prediction of the enriched term.

Ingenuity Pathway Analysis (IPA) software was used to compare transcriptomics data of endometrial samples collected from E2-treated animals (833 ng and 33.3 μg/infusion) with data generated for samples collected from gilts on day 12 of pregnancy and the estrous cycle. We found that among 65 canonical pathways that were activated or inhibited (Z-score higher than 2 or lower than −2 respectively) by pregnancy-specific DEGs, 13 were found to be affected also by E2 (33.3 μg/infusion). These were Glycoprotein VI Platelet (GP6) Signaling Pathway, Interleukin 8 (IL8) Signaling, Integrin Signaling, Leukocyte Extravasation Signaling, Actin Cytoskeleton, Signaling, Iintegrin-Linked Kinase Signaling, the Neuroinflammation Signaling Pathway, Fcγ Receptor-mediated Phagocytosis in Macrophages and Monocytes, Signaling by Rho Family GTPases, RhoA Signaling, Inhibition of Angiogenesis by Thrombospondin 1, the T Cell Exhaustion Signaling Pathway, and RhoGDI Signaling. Interestingly, 55 canonical pathways were identified to be exclusively activated or inhibited by E2-affected DEGs (i.e., peroxisome proliferator-activated receptor (PPAR) Signaling, Toll-like receptor signaling, ephrin receptor signaling, interleukin-6 (IL6) signaling, NF-κβ signaling, and the apelin endothelial signaling pathway). The most interesting canonical pathways affected by DEGs detected in analyzed groups are presented as a heatmap in Figure 5A. The complete results are summarized in Supplementary Table S9.

Analysis of diseases and bio functions revealed 64 processes related either to pregnancy-associated or E2-affected DEGs. The most interesting inhibited functions were related to apoptosis and organismal death, whereas the most interesting functions that were found to be activated were related with cellular homeostasis, angiogenesis, and the development of vasculature, fatty acid metabolism, migration of cells, and phosphorylation of proteins. We also found 80 processes exclusively related with E2-affected DEGs. The most interesting were related to angiogenesis and vasculogenesis, development of epithelial tissue, and the inflammatory response. A comparison of selected processes related with pregnancy-associated and E2-regulated DEGs is presented in Figure 5B. The complete results of diseases and bio functions analysis are summarized in Supplementary Table S10.

Upstream regulator analysis was used to predict molecules, including microRNAs and transcription factors, which may be causing the observed gene expression changes in the analyzed datasets. Indeed, among 306 molecules potentially affecting the expression of DEGs identified in endometrial samples on day 12 of pregnancy, 22 molecules were found to potentially affect DEGs regulated by E2. The most interesting upstream regulators common for DEGs detected in all analyzed groups were miR-16-5p (and other miRNAs w/seed AGCAGCA), prostaglandin E2 (PGE2), vascular endothelial growth factor A (VEGFA), interleukin 1β (IL1B), and tumor necrosis factor (TNF). Interestingly, up-stream analysis performed for pregnancy-associated DEGs revealed that these genes may be potentially regulated by E2 but also by other molecules commonly known to be involved in pregnancy establishment: PGE2, prolactin (PRL), transforming growth factors beta 1–3 (TGFB1-3), P4 and interferon gamma (IFNG), and interleukin 1B (IL1B); Supplementary Figure S5).

Similarly, *IFNG, TGFB1, TGFB2, PRL, PGE2, TNF*, and transforming growth factor alpha were found to potentially regulate E2-affected DEGs (Supplementary Figures S6 and S7). A comparison of selected molecules identified as potential upstream regulators of DEGs identified in analyzed groups are illustrated in Figure 5C. The complete results of the upstream analysis are summarized in Supplementary Table S11.

### 2.4. Validation of the Microarray Results by Quantitative Real-Time RT-PCR

The expression of the following DEGs identified in analyzed groups was validated using quantitative PCR (real-time RT-PCR): ATP binding cassette subfamily C member 4 (*ABCC4*); acyl-CoA synthetase long-chain family member 4 (*ACSL4*); ADAM metallopeptidase domain 9 (*ADAM9*); ADAM metallopeptidase with thrombospondin type 1 motif 20 (*ADAMTS20*); bradykinin receptor B2 (*B2R*); caspase 3 (*CASP3*); CCAAT/enhancer binding protein beta (*CEBPB*); claudin 1 (*CLDN1*); deleted in malignant brain tumors 1 (*DMBT1*); growth differentiation factor 15 (*GDF15*); heparin binding EGF like growth factor (*HBEGF*); interleukin 1 beta (*IL1B*); kelch like family member 14 (*KLHL14*); lysophosphatidic acid receptor 3 (*LPAR3*); mucin 4, cell surface associated (*MUC4*); serpin family B member 7 (*SERPINB7*); SMAD family member 3 (*SMAD3*); STEAP family member 1 (*STEAP1*); transient receptor potential cation channel subfamily V member 6 (*TRPV6*); Wnt family member 5A (*WNT5A*); and Wnt family member 7A (*WNT7A*).

**Figure 6:**
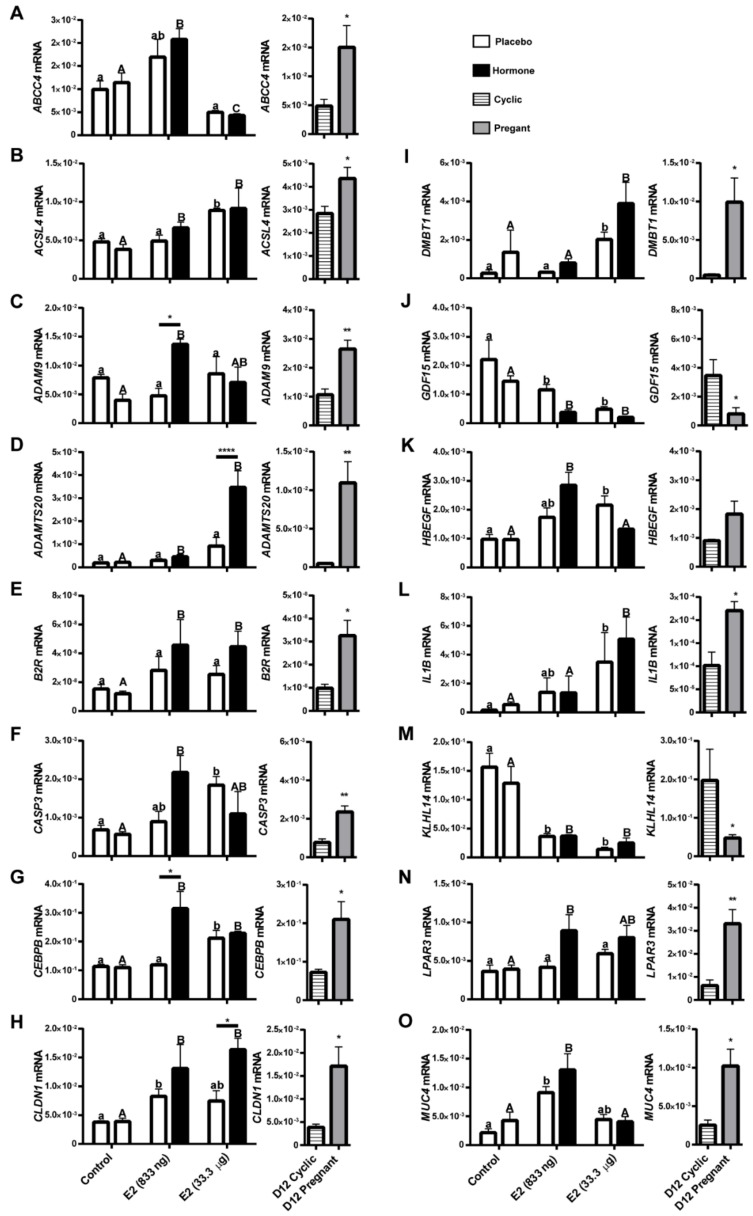

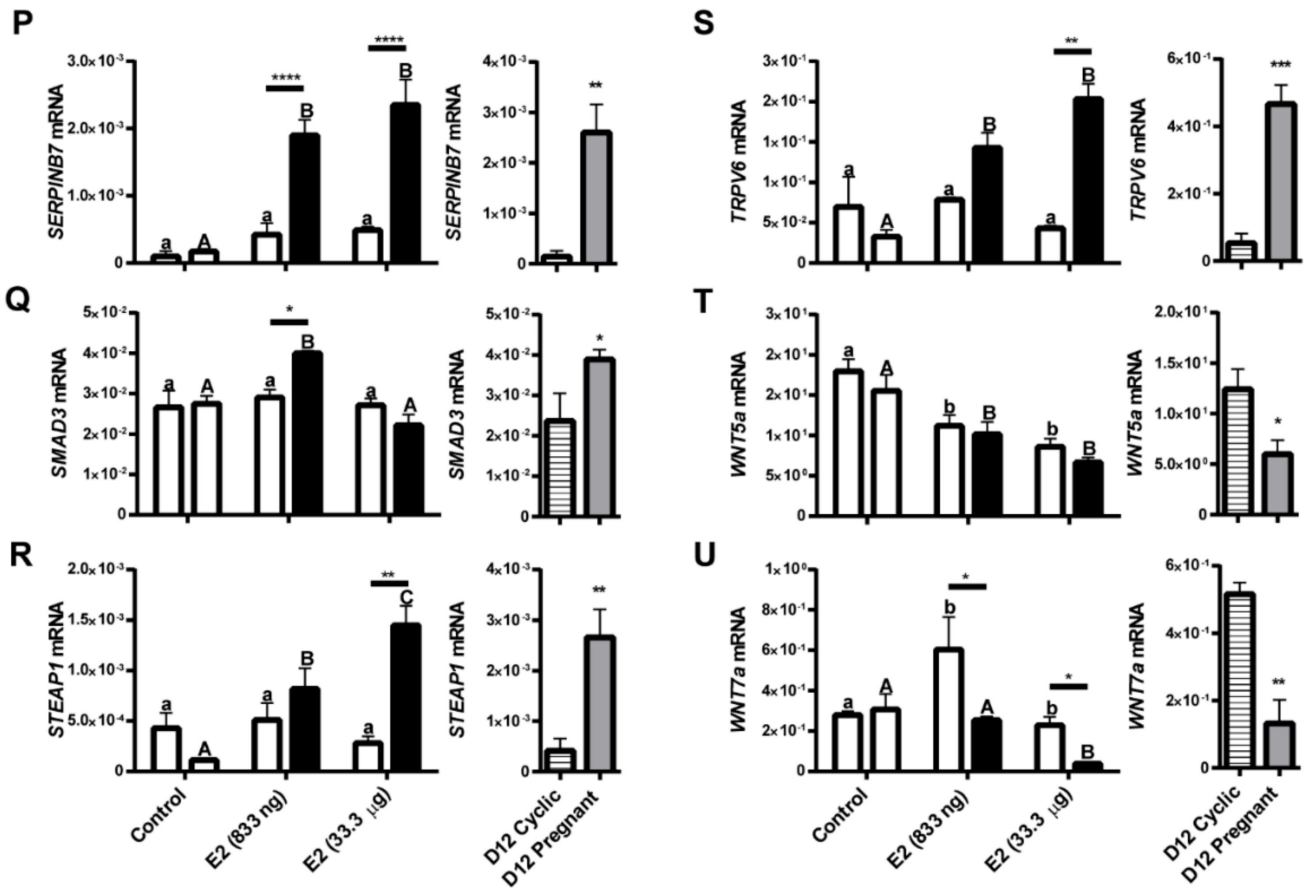
Expression of selected target genes determined by real-time RT-PCR in porcine endometrial samples treated with a placebo (control) or E2 (833 ng and 33.333.3 μg/infusion) and on day 12 of the estrous cycle (D12 Cyclic) and pregnancy (D12 Pregnant). Data are presented as the mean ± SEM. The expression of genes was normalized against the geometrical mean of RPL13A and GAPDH expression values. Different letters indicate statistically significant differences (two-way ANOVA, followed by the Bonferroni post-test; *p* < 0.05) in the local-indirect effect of E2 on gene expression. Asterisks (* *p* < 0.05; ** *p* < 0.01; *** *p* < 0.001; **** *p* < 0.0001) indicate the significant differences within the direct local effect of the E2 on gene expression. (**A**) ATP binding cassette subfamily C member 4 (*ABCC4*); (**B**) acyl-CoA synthase long-chain family member 4 (*ACSL4*); (**C**) ADAM metallopeptidase domain 9 (*ADAM9*); (**D**) ADAM metallopeptidase with thrombospondin type 1 motif 20 (*ADAMTS20*); (**E**) Bradykinin Receptor B2 (*B2R*); (**F**) caspase 3 (*CASP3*); (**G**) CCAAT/enhancer binding protein beta (*CEBPB*); (**H**) claudin 1 (*CLDN1*); (**I**) deleted in malignant brain tumors 1 (*DMBT1*); (**J**) growth differentiation factor 15 (*GDF15*); (**K**) heparin binding EGF like growth factor (*HBEGF*); (**L**) interleukin 1 beta (*IL1B*); (**M**) kelch like family member 14 (*KLHL14*); (**N**) lysophosphatidic acid receptor 3 (*LPAR3*); (**O**) mucin 4, cell surface associated (*MUC4*); (**P**) serpin family B member 7 (*SERPINB7*); (**Q**) SMAD family member 3 (*SMAD3*); (**R**) STEAP family member 1 (*STEAP1*); (**S**) transient receptor potential cation channel subfamily V member 6 (*TRPV6*); (**T**) Wnt family member 5A (*WNT5A*); and (**U**) Wnt family member 7A (*WNT7A*).

Overall, qPCR analysis confirmed the pattern of gene expression detected using expression microarrays in analyzed groups. *GDF15*, *KLHL14*, *WNT5a,* and *WNT7a* genes were found to be down-regulated (*p* < 0.001 – *p* < 0.05) both in endometrial samples treated with E2 and in endometrial samples collected on day 12 of pregnancy. *ADAM9, ADAMTS20, B2R, CASP3, CEBPB, CLDN1, DMBT1, IL1B, LPAR3, MUC4, SERPINB7, SMAD3, STEAP1,* and *TRPV6* genes were found to be up-regulated (*p* < 0.001 – *p* < 0.05) either in endometrial samples collected from in vivo models and in endometrial samples collected from gilts on day 12 of pregnancy (when compared to day 12 of the estrous cycle). The expression of *HBEGF* gene was found to be elevated in endometrial samples treated with E2 (833 ng/infusion; *p* > 0.001), but no difference was observed on day 12 of pregnancy vs. the estrous cycle (*p* > 0.05). The results of the microarray validation are summarized in Supplementary Table S12. The direct and indirect local effects of estradiol-17β on selected gene expression in porcine endometrium in vivo compared with their expression in endometrial samples collected on day 12 of the pregnancy and the estrous cycle are presented in Figure 6.

### 2.5. The Effects of E2 on PGE2 Secretion and PGF2α Metabolite (PGFM) Accumulation

**Figure 7:**
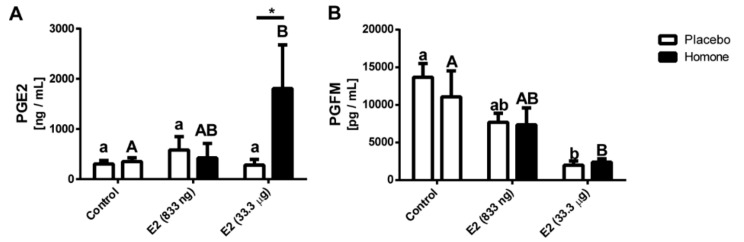
Concentration of prostaglandin E2 (PGE2) (**A**) and PGF2α metabolite (PGFM) (**B**) in the conditioned media form endometrial explants collected from gilts treated with a placebo (control) or E2 (833 ng and 33.3 μg/infusion) in vivo. Data are presented as the mean ± SEM. Different letters indicate statistically significant differences (two-way ANOVA, followed by Bonferroni post-test; *p* < 0.05) in concentration of PGE2 or PGFM in culture media. Asterisks * *p* < 0.05; ** *p* < 0.01; *** *p* < 0.001; **** *p* < 0.0001) indicate the significant differences within the direct local effect of the E2 on PGE2 or PGFM secretion by endometrial explants.

The highest concentration of PGE2 was detected in media collected after incubation of endometrial explants isolated from uterine horn treated with E2 (33.3 μg/infusion; *p* < 0.05; Figure 7A). An intermediate concentration of PGE2 was found in media collected after incubation of endometrial explants isolated from uterine horn treated with lower dose of E2 (833 ng/infusion) whereas the lowest PGE2 concentration was found in media collected from endometrial explants of gilts treated with placebo. No differences in PGE2 concentration were found for media collected after incubation of endometrial explants isolated from saline-treated horns.

Accumulation of PGFM (PGF2α metabolite) in media after culture of endometrial explants collected from both saline- and hormone-treated uterine horns of E2-treated gilts (33.3 μg/infusion) was decreased when compared to the control group (*p* < 0.05; Figure 7B).

## 3. Discussion

During early pregnancy porcine conceptuses secrete a pregnancy recognition signal (estradiol-17β; E2) required for prolonged progesterone synthesis and secretion by corpora lutea (CLs). Progesterone, in turn primes endometrial function enabling conceptus development and implantation. The present study is the first demonstrating the effect of E2 on the global gene expression profile in porcine endometrium in vivo during the time corresponding to the maternal recognition of pregnancy. We also compared the biological processes and functions related to E2-regulated genes with those associated with pregnancy-specific DEGs. The strength of the study is that the applied approach of in utero infusion of E2 is much closer to physiological conditions than the in vivo model of pseudopregnancy with systemic administration of E2 using high doses of E2 [16]. We found that a lower dose of E2 (833 ng/infusion) resulted in smaller alterations within the endometrial transcriptome, whereas a higher dose of E2 (33.3 μg/infusion) induced more changes in the global gene expression profile that were also similar to changes identified in day 12 of the pregnancy endometrial transcriptome. The limitation of the present study is the use of porcine expression microarrays for endometrial transcriptome profiling. With this technique the analysis is limited by the number of annotated genes at the time of the array design.

### 3.1. The Effect of Estradiol-17β on Porcine Endometrial Transcriptome In Vivo

Using expression microarrays, we determined the effects of E2 on the endometrial transcriptome in vivo and compared them with the effects of pregnancy on day 12 after estrus. Functional annotation clustering performed in DAVID revealed that E2-affected genes were enriched in high number of gene ontology terms related to metabolism (i.e., glucose metabolic processes and lipid metabolism), cellular processes (i.e., transmembrane transport, protein phosphorylation, cell division, and proliferation), tissue rearrangements (i.e., cell differentiation, focal adhesion, angiogenesis, and gland development), immune response (i.e., cytokine production and leukocyte migration), and many others. These findings stay in line with the results generated by DAVID for pregnancy-specific DEGs but also with previous studies on transcriptome changes in the porcine endometrium during early pregnancy [12,13].

Interestingly, the Pearson correlation of analyzed samples based on expression signals of identified DEGs revealed that endometria treated with E2 (833 ng and 33.3 μg/infusion) were clustered with endometria collected on day 12 from pregnant gilts, whereas samples treated with placebo (saline) were clustered together with endometria collected on day 12 of the estrous cycle. This indicates that the major changes in the endometrial transcriptome observed on day 12 of pregnancy are induced by E2.

The observation that E2 is a potent regulator of changes in the porcine endometrial transcriptome is consistent with our findings indicating that the higher dose of E2 resulted in more similarities in transcriptome changes found for day 12 of pregnancy when compared to the lower dose. Present results suggesting a critical role of E2 in pregnancy are also in agreement with the previous findings that premature exposure of pregnant gilts to exogenous estrogen (on days 9–10 of pregnancy) results in a significant alteration in the endometrial global gene expression profiles that likely causes desynchronization of the uterine environment and results in embryonic loss between days 15 and 18 of pregnancy [17].

On the other hand, E2 is not the only regulator affecting the endometrial transcriptome profile. Thus, we compared the functional annotation analysis results to determine which processes were enriched either by pregnancy-specific and E2-affected DEGs and to identify processes related with DEGs identified exclusively for day 12 of pregnancy or for the effect of E2 alone. Using the Topp Cluster software, we found multiple terms common for day 12 of pregnancy-specific and E2-regulated DEGs, which, likewise in DAVID, were related with processes involved in embryo–maternal interactions and preparation of the endometrial tissue for embryo implantation (i.e., integrin signaling, focal adhesion, angiogenesis, leukocyte migration, ion homeostasis, gland development, adherens junctions, and others). However, some processes were enriched only for E2-affected genes (i.e., HIF1A signaling and Toll like receptor cascades) or for pregnancy-associated DEGs (cytokine production by macrophages, protein digestion, and absorption).

Additional comparisons were performed using Ingenuity Pathway Analysis software, which, besides the identification of enriched terms, displays a prediction of activation or inhibition as a Z-score value. Overall, the results generated using IPA analysis were similar to those obtained using DAVID and Topp Cluster. However, the most interesting canonical pathway that was not identified using above mentioned tools was the PPAR signaling pathway, which was inhibited both in pregnancy and E2-treated endometrial samples. Among canonical pathways exclusively activated by E2, the ephrin receptor signaling pathway and apelin endothelial signaling pathway were identified. Interestingly, transcriptome profiling of porcine endometrium during the peri-implantation period revealed that the members of the EPH-ephrin system were significantly enriched for terms related to developmental processes [18].Based on data indicating the involvement of the EPH-ephrin system in regulation of the endometrial epithelial cell barrier [19], cell–cell and cell-matrix adhesion, and cell migration [20], it has been suggested that the EPH-ephrin system could be involved in a mechanism of control for trophoblast attachment and inhibition of invasion through the endometrial epithelium [18]. However, further studies on changes within the porcine endometrial transcriptome during the time of conceptus attachment revealed that only two members of EPH-ephrin system are expressed in the luminal epithelium (*EFNA1* and *EFNA5*), whereas in stromal cells and blood vessels only *EFNA1* expression has been detected [14].

Comparing the diseases and bio function activated by DEGs detected in pregnant and E2-treated animals were related to cell movement, cell adhesion, organization of the cytoskeleton, adhesion of immune cells, cell proliferation, fatty acid metabolism, angiogenesis, and others. On the other hand, we found that apoptosis was inhibited in the endometrium of pregnant and E2-treated animals.

### 3.2. Potential Upstream Regulators of DEGs

Analyzing the DEGs identified in endometrial samples of pregnant and E2-treated animals we found multiple potential upstream regulators. Interestingly, we found that DEGs identified in the endometrium of pregnant gilts may be potentially regulated by factors that have already been described to participate in pregnancy establishment: PGE2, TGFB1-3, IFNG, IL1B, PRL, and progesterone (reviewed in [2]). Intriguingly, a recent study suggested that E2 production by the conceptus is not required for pre-implantation conceptus development and elongation, as well as for early CL maintenance, but is essential for maintenance of pregnancy beyond 30 days [11]. On the other hand, results of upstream regulators performed in the present study indicate that the endometrial transcriptome changes triggered by E2 could be induced by other factors secreted by the conceptus and/or endometrium. Moreover, previous studies revealed that, in addition to porcine conceptuses, both the endometrium and myometrium could be other sources of estrogens in the porcine uterus [21]. It is then possible that E2 of endometrial origin together with other factors secreted and/or induced by conceptus signals (i.e., PGE2 [22]) may constitute compensatory mechanisms that are sufficient for maintenance of CL function and early pregnancy development in the pig. Our previous studies revealed that the profiles of PGE2 synthase expression and PGE2 secretion by porcine endometrium and conceptus are convergent to the profile of estradiol-17β secretion by porcine conceptuses [22]. As we identified PGE2 as a potential regulator of DEGs either in endometrial samples collected on day 12 of pregnancy and in endometrial samples treated with E2 in vivo, we speculate that PGE2, when together with estrogens of uterine origin and other factors secreting both by the endometrium and conceptuses, is sufficient to enable implantation of embryos and support CL function till day 30 of pregnancy. Therefore, findings from the functional analyses of microarray data indicate that the transcriptome changes should not rather be regarded as the effect of only one factor but, more likely, as a result of the interplay occurring between different hormones, cytokines, and other factors. Hence, further research on the effect of PGE2 and its synergistic action with E2 on global gene expression profile in vivo will be performed by our group.

### 3.3. Validation of the Microarray Results by Quantitative PCR

Based on the functional annotation of DEGs identified in the endometrium on day 12 of pregnancy and in the endometrium treated with E2 in vivo, we selected 21 genes whose expression was validated by quantitative PCR. Overall, a good agreement was found for the results obtained from the microarray and quantitative PCR. We found that E2 acting in a local-direct manner differentially regulated the expression of *ADAM9, ADAMTS20, CEBPB, CLDN1, SERPINB7, SMAD3, STEP1, TRPV6,* and *WNT7a* genes whereas *ABCC4, ACSL4, B2R, CASP3, DMBT1, GDF15, HBEGF, IL1B, KLHL14, LPAR3, MUC4, SMAD3, STEAP1, TRPV6,* and *WNT5a* were regulated by E2 acting in local-indirect manner (their expression was changed in hormone-treated and placebo–treated uterine horn of experimental group). It is consistent with earlier reports characterizing two types of effects of E2 infusions into uterine lumen in pigs—local and systemic [23]. Interestingly, the patterns of endometrial DEGs regulation by E2 were identical to the expression of DEGs detected in endometrial samples on day 12 of pregnancy. Here, we briefly discuss the importance of the most interesting DEGs regarding their involvement in the most important processes such as secretion, tissue remodeling, cell differentiation and proliferation, regulation of immune response, and other processes accompanying embryo implantation.

### 3.4. Estradiol-17β Affects the Expression of Genes Involved in Secretive Function and Ion Transport in Endometrium

Gene ontology terms related to secretion were significantly enriched in day 12 of pregnancy-associated and E2-affected DEGs. These results stay in line with previous findings that exogenous administration of estrogens on day 11 after estrus results in an increase in uterine luminal secretions of endometrial proteins, PGs and calcium [8]. Similarly, we evidenced that local infusion of E2 directly into the uterine lumen resulted in an elevated capacity of endometrial explants for secretion of PGE2. Moreover, a decreased content of PGFM was detected in culture media collected after incubation of endometrial explants treated with E2 in vivo. This result, however, does not necessarily mean that E2 inhibits PGF2α synthesis in porcine endometrium. Accumulation of PGF2α in porcine uterine lumen and its increased synthesis or PGF2α synthase in the endometrium on later days of pregnancy (day 15) during the implantation period has been reported [24,25,26]. Furthermore, endometrial expression of the 15-hydroxyprostaglandin dehydrogenase gene involved in prostaglandin catabolism (also in conversion of PGF2α to PGFM) has been found to be reduced on day 12 of pregnancy in pigs [13]. Results of the targeted gene expression validation revealed stimulating effect of E2 on *IL1B* and *ABCC4* gene expression. The importance of these genes in prostaglandin secretion has been reported in porcine endometrium [26,27]. Thus, present results confirm our previous findings on the importance of E2 as an embryonic signal regulating PGs synthesis and secretion [2,28].

Another group of enriched gene ontology (GO) terms contained genes involved in production and regulation of histotroph (e.g., transporter activity, calcium ion binding, and ion homeostasis). Histotroph, which includes transport proteins, enzymes, growth factors, and extracellular matrix proteins, acts on conceptus development, implantation, and placentation [29]. Since calcium ions are involved in the exocytotic process [30] they may act on exocytotic secretion of various histotrophic molecules in the uterine endometrium.

The calcium balance in mammalian cells is essential for many physiological functions including cellular processes such as gene regulation, cell differentiation, and apoptosis [31]. In humans, binding endometrial epithelial cells to trophoblast cells results in a calcium influx into endometrial epithelial cells [32]. Concentration of calcium ions in porcine uterine lumen is increased during the periimplantation period [8]. Our results are consistent with reports indicating a key role of calcium in the attachment of the conceptus to endometrium. Calcium facilitates cell-to-cell and extracellular matrix (ECM) adhesion, as many cell adhesion molecules such as integrins, cadherins, selectins, and ECM proteins require calcium ions for their activity [33,34]. Quantitative analysis of gene expression revealed that E2 induced expression of *TRPV6* gene that mediates calcium ion uptake [35]. Our data confirmed previous findings concerning regulation of TRPV6 by E2 and its up-regulation on day 12 of pregnancy [36]. TRPV6 was suggested to be a marker for implantation in the porcine endometrium [36]. In murine embryos TRPV6 is responsible for calcium ion homeostasis [37], whereas silencing its expression in human trophoblast cells resulted in diminished cell proliferation and induced apoptosis [38]. Interestingly, among the top five up-regulated genes in E2-treated and day 12 of pregnant endometrium were other genes related to calcium binding *S100A8*, *S100A9*, and *S100A12*. S100A8 and S100A9 have been described to act as survival factors for early-stage murine embryos [39]. Moreover, up-regulation of *S100A8* gene expression has been found in the porcine endometrium on day 12 and 14 of pregnancy [13,18]. In cattle, *S100A8* and *S100A9* genes were associated with inflammatory changes of endometrial tissue. *STEAP1,* which is related to ion homeostasis and ion transport, is another gene strongly up-regulated by E2 and on day 12 of pregnancy in porcine endometrium, STEAP1 has been described to be involved in intercellular communication [40,41]. Thus, our results indicate that E2, as the primary embryonic signal, is also responsible for ion homeostasis, which is required for other processes involved in the establishment of pregnancy in the pig.

Extracellular vesicles (EVs)/exosomes were the most enriched gene ontology terms related with day 12 of pregnancy-specific and E2-affected DEGs. These structures are emerging as novel regulators of interactions occurring at the embryo–maternal interface. The importance of EVs and their cargo in regulation of endometrial function and conceptus growth during pregnancy establishment has been described in humans [42], pigs [43], and sheep [44].

### 3.5. Estradiol-17β Affects Processes Related with Immune Response

Implantation and establishment of pregnancy are associated with a highly coordinated increase in the expression of inflammatory mediators at the embryo–maternal interface. During the peri-implantation period, various inflammatory mediators such as E2, interferons, interleukins, and prostaglandins create a proinflammatory environment (reviewed in [2,4]). Interestingly, our microarray data suggest that E2 during early pregnancy in the pig is involved in shifting towards the Th1 pathway from Th2, a common phenomenon occurring during implantation period in other species [4]. Functional analyses of transcriptome data of endometrial samples collected on day 12 of pregnancy and from in vivo models revealed high enrichment of terms related with immune response and immunotolerance.

Gene expression analyses confirmed up-regulated expression of *IL1B* both on day 12 of pregnancy and in E2-treated endometrial samples. The elevated expression of IL1B has been detected in porcine conceptuses during morphological transformation to filamentous form [45]. At the porcine embryo-maternal interface, IL1B has been suggested to modulate the proinflammatory reaction of the uterus during elongation and placental attachment (reviewed [46]). 

Our results are consistent with transcriptome profiling performed in porcine endometrium and in LE and GE, which revealed *SERPINB7* as one of the most up-regulated genes on day 12 and 14 of pregnancy [13,14,18]. The role of SERPIN family proteins in immunomodulation in reproductive tissues have been described in cattle [47].In the present study we evidenced the local-direct highly stimulating effect of E2 on expression of *SERPINB7* that may suggest its contribution into immune interactions at the embryo-maternal interface during pregnancy establishment in pigs. In mice endometrium, SERPINB7 was localized mainly in luminal epithelial cells and it has been suggested that its increased levels may inhibit protease (plasmin) activity that normally leads to extracellular matrix degradation [48]. Up-regulated expression of SERPINB7 in response to E2 treatment may suggest its contribution to ECM remodeling during the pre-attachment phase of pregnancy in pigs.

One of the down-regulated genes on day 12 of pregnancy and in the E2-treated endometrium was *KLHL14* gene, a member of the Kelch-like gene family, whose members contain a broad-complex tramtrack and bric a brac (BTB, also known as Poxvirus and Zinc finger—POZ) domain, a BACK domain, and several Kelch domains [49]. The BTB/POZ domain facilitates protein binding (reviewed in [50]). Other BTB-containing proteins were found to be involved in a variety of cellular mechanisms i.e., control of cytoskeletal organization [51], ion channel gating [52], or transcription suppression [53]. In human endometrium expression, the *KLHL14* gene was found to be up-regulated during the secretory phase of the menstrual cycle [54]. Interestingly, studies performed in mice suggest that KLHL14 promotes lymphocytes B-1a development in mice [55]. Hence, we hypothesize that down-regulation of *KLHL14* by E2 may modulate the immune response of endometrial tissue during early pregnancy.

### 3.6. Involvement of Estrogen Signaling in Processes Related to Cell Adhesion, Differentiation, and Proliferation and Tissue Remodeling

Adhesion of trophoblast cells to the epithelial layer of the endometrium precedes the embryo implantation in all mammalian species (reviewed in [56]) and was one of the GO terms enriched in our results and related to genes such as *PTGER2*, *CLDN1,* and *MUC4.* Hence, a profound rearrangement of endometrial tissue is required for successful implantation of embryos. The remodeling of endometrial epithelium resulting in changes of its structure, polarity, and adhesive properties is irrespective of the type of placenta and has been described in humans as well as in animal species [57,58]. Previously, we reported that PGE2 acting through its receptor (PTGER2), regulated adhesion of the porcine and human trophoblast to ECM proteins [59]. Results from transcriptome profiling performed in the present study revealed that expression of PTGER2 was stimulated by E2, in line with our previous studies [28]. 

Claudins are a family of integral membrane proteins contributing to both adhesion and barrier properties of tight junctions [60]. During the receptive phase, the apico-basal polarity of endometrial epithelial cells is reduced and accompanied with redistribution of cell-cell junction proteins to facilitate apposition between the embryo and the endometrium [61]. Interestingly, recent studies revealed the up-regulated expression of the *CLDN1* gene and protein in porcine endometrium on day 12 of pregnancy [62].

Mucin 4 is a high-molecular-weight glycoprotein proposed to protect the surface of most epithelia [63]. Its expression in porcine surface and glandular epithelia increased in the luteal phase and declined during early proestrus [64]. Additionally, the expression of MUC4 was found to be elevated on days 12 and 14 of pregnancy when compared to days 12 and 14 of the estrous cycle [12,13]. Therefore, it has been speculated that it is involved in protecting the epithelial layer of the endometrium against invasive trophoblasts. Accordingly, in the present study we confirmed the previous findings and we also evidenced that this up-regulation of MUC4 gene is related with E2 signaling.

Other group of molecules that were up-regulated both by E2 and on day 12 of pregnancy belong to a disintegrin and metalloprotease (ADAM) family of membrane-anchored glycoproteins. Our results are consistent with previous reports indicating that ADAM9 might play an important role in the remodeling of the mouse uterus and rabbit endometrium during the peri-implantation period [65,66]. ADAMTS20 is a member of another ADAM family containing the thrombospondin motif [67]. The role of ADAMTS20 is related to cleaving ECM proteins (reviewed in [68]). However, its role in the reproductive processes in mammals is not specified.

Another E2-specific and pregnancy-specific DEG, also related in tissue remodeling, is macrophage inhibitory cytokine-1 (*MIC-1*), also named growth differentiation factor 15 (*GDF15*), a member of the transforming growth factor-β (TGFB) superfamily, known to be expressed at high levels in human placenta [69]. GDF15 has been reported to be involved in actin cytoskeleton reorganization and remodeling [70] and is necessary for the maintenance of pregnancy in humans [71] and rats [72]. Intriguingly, it has been suggested that GDF15 participates in placenta development via promoting trophoblast cell invasion [73]. Thus, the down-regulation of *GDF15* in porcine endometrium in response to E2 treatment may be due to a non-invasive type of implantation occurring in pigs.

Another gene related to TGFB, which was DEG identified in both E2-treated and pregnant endometrium, was SMAD3, which belongs to the main signal transducers for receptors of the TGFB superfamily. Its activation and subsequent translocation into the nucleus results in gene expression in response to TGFB [74]. Thus, it can be suggested that E2-stimulated expression of endometrial *SMAD3* may serve as the mechanism sustaining the TGFB signaling in porcine endometrium during early pregnancy therefore contributing to enhanced cell differentiation and proliferation and regulation of porcine trophoblast adhesion and invasiveness [75,76].

Changes within the endometrial structure also involve processes related to cell proliferation and differentiation. CCAAT/enhancer-binding protein beta (CEBPB) has been described as a critical mediator of steroid hormone-regulated cell proliferation and differentiation and steroid hormone responsiveness in the uterine epithelium and stroma in mice [77]. In humans the expression of CEBPB was detected in at the implantation sites and has been described as a marker of uterine receptivity [78]. In the present study we found a direct local effect of E2 on the elevated expression of *CEBPB* in porcine endometria. Thus, its role in endometrial receptivity during early pregnancy in the pig requires further investigation. Another factor related with cell proliferation and differentiation is DMBT1. Studies on both primate and rodent uteri demonstrated that DMBT1 is an E2-induced protein and was localized to the epithelial layer of the endometrium. Moreover, it has been suggested that it may be involved in endometrial growth and/or differentiation [79]. Accordingly, we demonstrated that the expression of *DMBT1* is up-regulated in porcine endometrium on day 12 of pregnancy and that this up-regulation is induced by E2.

The results of the present study showing the up-regulation of endometrial *LPAR3* expression on day 12 of pregnancy are in line with elevated LPAR3 expression in porcine endometria [80]. Interestingly, we demonstrated that this expression is regulated by E2 signaling in vivo. LPAR3 is considered to be a uterine receptivity marker critical for embryo migration and spacing in mice [81]. The LPA-LPAR3 signaling may be involved in the development of trophoblasts during early pregnancy in pigs, as it was evidenced that LPA promoted proliferation, migration, and differentiation of porcine trophoblast cells by activating the ERK1/2-P90RSK-RPS6 and P38 pathways [82]. Moreover, LPA can regulate PG synthesis in bovine endometrium [83].

Intriguingly, results generated by Ingenuity Pathway Analysis software revealed that E2-regulated genes were enriched with terms related to cancer diseases. Indeed, endometrioid type endometrial cancers (EEC), which represent around 70–80% of all endometrial cancers, are associated with increasing estrogen levels that, in turn are responsible for impaired control of cell proliferation (reviewed in [84]). Using the Hec-1A endometrial cancer cell line it has been demonstrated that diacylglycerol kinase alpha (DGKA) activity is required for E2-stimulated cell proliferation, as using DGKA inhibitors resulted in E2-stimulated proliferation and growth of Hec-1A cells [85]. Interestingly, the gene expression of iota and delta isoforms of diacyloglicerol kinase (*DGKI*, *DGKD*) have been found to be down-regulated in E2-treated and day 12 of pregnancy endometrial samples. However, unlike in cancer tissue, the growth of the endometrium during pregnancy is limited. Transcriptome profiling revealed that E2 (33.3 μg/infusion) lead to down-regulation of insulin growth factor 1 (*IGF1*), a cytokine that has been suggested to promote cancer survival and tumor metastasis [86,87]. Hence, the above-mentioned literature data together with our results from endometrial transcriptome profiling suggest the existence of mechanisms controlling the E2-induced changes within endometrial tissue during pregnancy.

Summarizing, present study is the first showing the effect of E2 on the global gene expression profile in porcine endometrium in vivo, in the period corresponding to maternal recognition of pregnancy. Using in vivo models involving physiological levels of E2 mimicking action of conceptus signal, we demonstrated that endometrial transcriptome changes induced by E2 are similar to those observed on day 12 of the pregnancy. Our results indicate that administration of estradiol-17β to the uterus results in hypertrophy of the surface epithelium, stromal cell differentiation, and an increase in glandular secretory activity, which are early indicators of maternal response to pregnancy. We also showed that E2, by regulation of gene expression in porcine endometrium, is a key factor triggering multiple processes, such as secretive function, secretion of extracellular vesicles, ion homeostasis, cell adhesion, immune response, tissue remodeling, and cell proliferation and differentiation that are required for successful implantation of embryos and development of early pregnancy. Moreover, our results suggest that changes observed in the endometrial transcriptome triggered by E2 can also be potentially controlled by other factors of both conceptus and maternal origin. The genes and pathways identified in the present study not only add to our understanding of the complex gene regulation dynamics regulated by E2 in the endometrium but also provide valuable resources for further targeted studies considering the importance of embryo–maternal interactions in successful implantation and pregnancy development.

## 4. Materials and Methods

All procedures involving the use of animals were conducted in accordance with the national guidelines for agricultural animal care and were approved by the Animal Ethics Committee, University of Warmia and Mazury in Olsztyn, Poland, permission No. 17/2008 (22 January 2008).

### 4.1. Tissue Collection

Prepubertal crossbred gilts of similar age (6 months) and genetic background were observed for the onset of the estrous cycle. After two natural estrous cycles, gilts were divided into two groups: cyclic and pregnant. Cyclic pigs were slaughtered in the local abattoir on day 12 of the estrous cycle (n = 6 per every group) whereas pregnant gilts were slaughtered on day 12 of pregnancy (n = 6 per every group). Pregnancy was confirmed by the presence of conceptuses. The conceptuses were flushed from each uterine horn with sterile phosphate-buffered saline (PBS; 137 mM NaCl, 27 mM KCl, 10 mM Na2HPO4, and 2 mM KH2PO4, pH 7.4). Endometrial fragments from collected uteri were dissected from myometrium at the middle part of uterine horn. In pregnant gilts, the endometrium was also separated from trophoblasts and was collected from implantation sites. Endometrial samples were snap-frozen in liquid nitrogen and stored in −80 °C for further analyses.

### 4.2. Effect of E2 on Porcine Endometrium In Vivo

The effects of E2 administered directly into the uterine lumen was studied using in vivo models [88]. Prepubertal crossbred gilts of similar age (6 months) and genetic background after the second natural estrous cycle were treated hormonally with 750 I.U. PMSG (Folligon, Intervet, Boxmeer, The Netherlands) and 500 I.U. of hCG (Chorulon, Intervet) given 72 h later to induce estrus. Between days 12–14 of the estrous cycle, gilts were injected with 10 mg PGF2α (Dinolytic; Pfizer, Warsaw, Poland). Sixteen hours later, 10 mg of PGF2α was injected simultaneously with 750 IU PMSG (Folligon; Intervet). After 72 h, 500 I.U. hCG (Chorulon; Intervet) was given intramuscularly. On days 8–9 of the third estrous cycle animals underwent surgery in which the cannula was introduced into the uterine lumen at distance of 10–15 cm from the isthmus.

To simulate hormone delivery by conceptuses the cannula was perforated along its length as reported previously [23] with some modifications. Animals were divided into three groups. In the control group, each horn received intrauterine placebo (5 mL of 1% *v/v* ethanol saline) infusions. In the experimental groups, the doses of hormones used were similar to those previously published [23]. Randomly selected horns within each gilt received hormonal infusions: E2 (833 ng or 33.3 µg/infusion) while the contralateral horn received only placebo infusions. Treatments were administered every 4 h for 24 h on days 11–12 after the onset of estrus. After the experiment, endometrial samples were collected as described in paragraph 4.1. Uteri with inflammatory changes and/or with fluid accumulation were eliminated from further procedures.

### 4.3. RNA Isolation and cDNA Synthesis

Total RNA was isolated from endometria using TRIzol (Invitrogen, Carlsbad, CA, USA) according to the manufacturer’s protocol. Purity and concentration of isolated RNA was measured with a NanoDrop spectrophotometer (Thermo Fisher Scientific, Waltham, MA, USA). Additionally, RNA integrity was assessed by using Agilent 2100 Bioanalyzer (Agilent Technologies, Santa Clara, CA, USA). To generate cDNA for the quantitative PCR (qPCR) reaction, the total RNA sample (1 µg) was reverse-transcribed with MultiScribe™ Reverse Transcriptase kit (Thermo Fisher Scientific) according to the manufacturer’s protocol. cDNA samples were stored in −80 °C for further qPCR analyses.

### 4.4. Global Gene Expression Profiling Using Expression Microarrays

Microarray analysis was performed using 100 ng total RNA isolated from endometrial tissues. Cy3-labeled cRNA was produced with the Low-Input Quick Amp Labeling Kit, one-color (Agilent Technologies) and hybridized to the Agilent 4x44k Porcine Gene Expression microarrays (G2519F-026440) according to the instructions provided by the manufacturer. Hybridized and washed slides were scanned at 2-μm resolution with an Agilent DNA Microarray Scanner (G2505C; Agilent Technologies). Image processing was performed with Feature Extraction Software 10.5.1.1 (Agilent Technologies). Signals were filtered based on “well above background” flags (detection in three of four samples) and normalized with the BioConductor package VSN [89].

For quality control, normalized data were analyzed with a distance matrix and a heatmap based on pair-wise distances (BioConductor package Geneplotter). Probes exhibiting significant differences in signal intensity were identified in the contrasts (pairwise comparison) “pregnant” vs. “cyclic” (endometrial samples collected from gilts on day 12 of pregnancy and the estrous cycle) and “hormone-treated horns” (experimental group) vs. “placebo-treated horns” (control group) using the BioConductor package LIMMA (Linear Model for Microarray Analysis; [90]). The following cut-offs were applied: log fold change > 0.585 and adjusted *p*-value < 0.05. The “FDR”-method (False Discovery Rate; FDR 5%, i.e., adjusted *p*-value < 0.05) was applied for the correction of multiple testing.

To visualize the distribution of expression signals of identified probes among samples, hierarchical clustering using the Pearson correlation was performed using Multiexperiment Viewer software (MeV; [91]). Identified significant probes were annotated based on mapping of the probe sequences (60 nt sequences) to the porcine genome (*SusScrofa* 11.1). Known and potential human ortholog or homolog genes were assigned using a ortholog annotation database (Mammalian Ortholog and Annotation Database, MAdb; [92]). If a gene in the list was represented by more than one probe sequence, the mean of log fold-change, *p*-value, and adjusted *p*-value (FDR) were calculated.

#### 4.4.1. Functional Annotation of Microarray Data

Lists of DEGs generated by LIMMA analysis were used as input data for functional annotation. To study the changes in global gene expression profile in endometrial samples on day 12 of the estrous cycle and pregnancy and the effect of E2, open-source and commercial software was applied. The Database for Annotation, Visualization, and Integrated Discovery (DAVID; [93,94]) was used to calculate the fold enrichment of identified gene ontology (GO) terms using the following databases: Functional Categories (UP_KEYWORDS); gene ontology Biological Process (GOTERM_BP_FAT), Cellular Component (GOTERM_CC_FAT); Molecular Function (GOTERM_MF_FAT); General Annotations (SP_COMMENT); Pathways (BIOCARTA; KEGG PATHWAY); Protein Domains (INTERPRO); and Protein Interactions (UCSC_TFBS). The results generated by DAVID were summarized in a tabular format.

To compare transcriptome changes associated with pregnancy with these induced by E2 treatment the expression signals of DEGs identified in endometrial samples collected from gilts on day 12 of pregnancy and the estrous cycle were matched with expression signals of genes in datasets generated for endometrial samples collected from in vivo model. Likewise, the expression signals of DEGs identified in endometrial samples treated with E2 (833 ng/infusion or 33.3 μg/infusion) were matched with expression signals of genes in dataset generated for endometrial samples collected from gilts on day 12 of pregnancy and the estrous cycle. Generated matrix of matched expression signals was then analyzed by hierarchical clustering using Pearson correlation. Common and group-specific DEGs were identified using jVenn plug in (jvenn.toulouse.inra.fr; [95]) and summarized in a Venn diagram and tabular format.

Topp Cluster software [96] was used to identify gene ontologies enriched by DEGs identified in endometria on day 12 of pregnancy (when compared to day 12 of the estrous cycle) and in endometrial samples treated with E2 (when compared to placebo). Results were summarized in a tabular format and visualized by interaction network.

Ingenuity Pathway Analysis (IPA; v. 01.12; Qiagen, Redwood City, CA, USA) software was used to compare canonical pathways, upstream regulators and bio functions associated with pregnancy-specific and E2-affected DEGs. Terms with prediction Z-score higher than 2 or lower than −2 may be regarded as significant [97]. The results of the performed analyzes were summarized in tabular and in graphic formats.

#### 4.4.2. Validation of Microarray Results

To verify the results obtained in microarray analysis the expression of the following genes was determined by qPCR (real-time RT-PCR): *ABCC4*; *ACSL4*; *ADAM9*; *ADAMTS20*; *B2R*; *CASP3*; CEBPB; *CLDN1*; *DMBT1*; *GDF15*; *HBEGF*; *IL1B*; *LPAR3*; *MUC*4; *SERPINB7*; *SMAD3*; *STEAP1*; *TRPV*6; *WNT5A;* and *WNT7A*. Real-time RT-PCR was performed using TaqMan assays (Thermo Fisher Scientific; accordingly to the manufacturer’s protocol or using oligonucleotide primers and SYBR Green (Thermo Fisher Scientific); Supplementary Table S13). Briefly, reverse-transcribed cDNA (3.5 µL) was added to the reaction mixture: 12.5 µL Power SYBR Green master mix, 2.5 µL of each sense and antisense primer (1 µM; Supplementary Table S12), and 4 µL of nuclease-free water.

The PCR programs were performed as follows: initial denaturation (95 °C, 10 min) followed by 36 cycles of denaturation (95 °C; 15 s), annealing and elongation (60 °C; 1 min). After PCR using SYBR Green, melting curves were acquired by gradual increases in the temperature from 60 to 95 °C to ensure that a single product was amplified in the PCR reaction. All real-time PCR reactions were performed with Applied Biosystems 7900HT Real-Time PCR system (Life Technologies, Carlsbad, CA, USA). Gene expression was estimated using real-time PCR Miner software [98]. Stability of the reference genes in the porcine endometrium was assessed using the statistical algorithm Normfinder 2.0 [99].

Six reference genes were analyzed: *ACTB, GAPDH, HMBS, HPRT1, PPIA,* and *RPL13A.* For normalization of results, the geometrical mean of the most stable pair of reference genes (*RPL13A* and *GAPDH*) was used. The direct local effect of E2 on endometrial gene expression was studied by comparison of mRNA levels in the endometrial samples from the hormone-treated horn to its expression in the samples from the placebo-treated horn of each gilt. The local indirect effects of E2 on endometrial gene expression were studied by comparison of mRNA levels in endometrial samples from the experimental group to the levels in the control group (vehicle infusions into both horns).

### 4.5. PGs Secretion by Endometrial Explants in Response to Estradiol-17β In Vivo

#### 4.5.1. Isolation of Endometrial Explants

The effect of E2 administered into uterine lumen in vivo on the capacity of secretion of PGE2 and PGF2α was studied in vitro. Briefly, endometrial explants were isolated as described previously [28]. Isolated endometrial tissue fragments were preincubated in medium 199 (M199, Sigma-Aldrich, St. Louis, MO, USA) supplemented with BSA (1%, wt/vol), newborn calf serum (NCS, 5% vol/vol), and antibiotics (penicillin/streptomycin, Sigma-Aldrich) for 2 h in humidified atmosphere of air and CO_2_ (5%) at 37 °C with gentle shaking. After preincubation, endometrial explants were flushed two times with sterile phosphate buffered saline (PBS) and placed in the vials containing incubation medium (M199 with antibiotics). Explants were then incubated for 24 h in humidified atmosphere of air and CO_2_ (5%) at 37 °C with gentle shaking. Following incubation, media were collected and stored at −80 °C until further analyses.

#### 4.5.2. Measurement of Hormone Concentration in Incubation Media

The content of secreted PGE2 and PGFM (PGF2α metabolite) in media collected after incubation of endometrial explants isolated from gilts after the in vivo experiment was determined using an enzyme immunoassay, as described previously [22]. Briefly, cross-reactivities of the anti-PGE2 antiserum (donated by Dr Seiji Ito, Kansai Medical University, Osaka, Japan) were as follows: 18% PGE1, 10% PGA1, 4.6% PGA2, 6.7% PGB2, 0.13% PGD2, 2.8%, PGF2α, 14% PGJ2, and 0.05% 15-keto-PGE2. Assay sensitivity was 0.19 ng/mL and the intra- and inter-assay coefficients of variation were 6.5% and 13.9% respectively. Concentrations of PGFM were determined using horseradish peroxidase-labeled PGFM and anti-PGFM antiserum (WS4468-7; donated by Dr William Silvia, University of Kentucky, Lexington, KY, USA). Cross-reactivities of the anti-PGFM antiserum with PGE2, PGA2, PGF2α, and 6-keto-PGF1 were lower than 0.1%. Assay sensitivity was 50 pg/mL and the intra- and inter-assay coefficients of variation were 6.6% and 11.2% respectively

### 4.6. Statistical Analyses

The T-test was used to determine statistical significance of gene expression difference in porcine endometrium on day 12 of pregnancy when compared to day 12 of the estrous cycle. Two-way ANOVA followed by the Bonferroni post-test was used to analyze the effect of E2 on gene expression and PG secretion in endometrial samples collected from in vivo experiments. Differences were considered as statistically significant at the 95% confidence level (*p* < 0.05). All statistical analyses were conducted using GraphPad PRISM v. 6.0 software (GraphPad Software Inc., San Diego, CA, USA).

## Figures and Tables

**Table 1:** Selected results Database for Annotation, Visualization, and Integrated Discovery (DAVID) functional annotation clustering for differentially expressed genes in porcine endometrium.

Selected Functional Terms of Overrepresented Annotation Clusters	Enrichment Score
**E2 – 833 ng/infusion**	
**UP REGULATED**	
extracellular exosome (45; 1.85), extracellular vesicle (45; 1.84)	3.76
glucose metabolic process (8; 5.41)	2.60
calcium ion transport (9; 3.14), ion homeostasis (16; 2.95), transmembrane transport (21; 2.05)	2.31
protein phosphorylation (28; 1.95)	2.07
positive regulation of cell growth (6; 5.02)	1.89
epithelial cell differentiation (12; 2.75)	1.79
cell adhesion (23; 1.74)	1.72
response to hypoxia (6; 2.71)	1.51
regulation of cell migration (11; 2.07)	1.45
placenta development (5; 4.45)	1.43
regulation of blood circulation (7; 3.01)	1.42
**DOWN REGULATED**	
extracellular exosome (23; 1.65), extracellular vesicle (23; 1.64)	1.78
steroid metabolic process (5; 3.59), lipid metabolism (6; 2.88)	1.66
protein ubiquitination (12; 3.09)	1.65
calcium transport (3; 6.36), sodium ion transport (5, 4.98), active transmembrane transporter activity (7, 3.60)	1.59
**E2 – 33.3 μg/infusion**	
**UP REGULATED**	
extracellular exosome (338; 1.85) extracellular vesicle (339; 1.85)	30.04
focal adhesion (77; 3.03), cell junction (143; 1.60)	13.69
regulation of inflammatory response (56; 2.96)	12.95
cell migration (135; 1.89)	9.57
cell proliferation (177; 1.58)	8.04
apoptotic process (177; 1.62)	7.67
positive regulation of mapk cascade (58; 2.02), activation of mapk activity (21; 2.39)	7.62
angiogenesis (61; 2.45)	7.32
cytokine production (75; 1.98)	6.77
leukocyte migration (59; 2.61)	5.70
lipid metabolism (57; 2.09)	4.72
gland development (54; 2.13)	4.37
response to hypoxia (38; 2.21)	3.77
cell division (64; 1.84), mitotic cell cycle (96; 1.64), regulation of cell cycle (81; 1.38)	3.54
maternal process involved in female pregnancy (11; 3.10), female pregnancy (28; 2.49); multi-organism reproductive process (70; 1.24)	3.37
ecm-receptor interaction (20; 2.79), extracellular matrix component (19; 2.28), extracellular matrix (27; 2.01)	3.12
protein transport (153; 1.37), intracellular protein transport (78; 1.28)	3.09
protein processing (37; 2.61), protein maturation (40; 2.45)	3.03
cellular response to interferon-gamma (20; 2.46)	3.02
epithelial cell proliferation (39; 1.87)	2.57
endothelium development (19; 2.84), endothelial cell differentiation (14; 2.45)	2.54
steroid hormone receptor binding (15; 3.10), hormone receptor binding (21; 2.29), nuclear hormone receptor binding (18; 2.28)	2.13
**DOWN REGULATED**	
cell development (111; 1.31), regulation of cell differentiation (88; 1.33)	4.06
cytoskeletal protein binding (59; 1.59)	2.84
cell migration (67; 1.32)	2.32
histone acetylation (16; 2.57)	2.05
import into cell (10; 3.28)	1.74
negative chemotaxis (8; 4.54)	1.67
**Day 12 of pregnancy vs day 12 of the estrous cycle**	
**UP** **REGULATED**	
vasculature development (54; 3.12)	11.26
cell migration (76; 2.26)	9.78
extracellular exosome (464; 1.51), extracellular vesicle (464; 1.50)	6.67
mitotic cell cycle (57; 2.02), cell cycle process (64; 1.66)	5.65
regulation of signal transduction (116; 1.50)	5.32
focal adhesion (32; 2.56 ), adherens junction (42; 1.89)	4.81
regulation of protein phosphorylation (67; 1.81)	4.75
tissue migration (22; 3.27), epithelial cell migration (19; 2.93)	4.55
response to hypoxia (22; 2.71)	4.04
activation of innate immune response (18; 2.53)	3.28
endothelial cell development (9; 5.37), endothelium development (11; 3.49)	3.12
immune response-regulating signaling pathway (28; 1.86)	2.26
immune system development (39; 1.67)	2.08
in utero embryonic development (20; 2.13)	1.84
**DOWN REGULATED**	
oxidation-reduction process (25; 2.31), organic acid metabolic process (21; 2.11), fatty acid metabolic process (11; 2.73), lipid metabolism (11; 2.63), lipid biosynthesis (5, 3.31) fatty acid biosynthesis (3; 5.97)	2.13
regulation of extent of cell growth (7; 6.12)	1.95
regulation of cell morphogenesis (16; 2.56), regulation of cell differentiation (30; 1.77), regulation of anatomical structure size (13; 2.44)	1.92
response to calcium ion (6; 4.63)	1.89
circulatory system development (19; 1.83)	1.62
response to hormone (16; 1.70)	1.60
regulation of transport (29; 1.47)	1.36

## Supplementary Materials

Supplementary materials can be found at https://www.mdpi.com/1422-0067/21/3/890/s1.
