# Supplementary material for: Estradiol-17β-Induced Changes in the Porcine Endometrial Transcriptome In Vivo
Source: Int J Mol Sci. 2020 Jan 30;21(3):890. doi: 10.3390/ijms21030890 (PMC7037416; doi:10.3390/ijms21030890)
Supplement: Supplementary file 1 [file ijms-21-00890-s001.zip › SUPPLEMENTARY FIGURE LEGENDS.docx]

**SUPPLEMENTARY FIGURE LEGENDS**

**Supplementary Figure 1.** Gene expression distance matrix of analyzed samples generated in Geneplotter library performed for comparisons: E2 (833 ng/infusion) vs placebo (A), E2 (33.3 μg/infusion) vs. placebo (B) and 12 day of pregnancy vs. 12 of the estrous cycle (C). VSN-normalized microarray expression data was clustered based on pair wise correlation using all detectable probes. CTRL – endometrial samples collected from gilts assigned to control groups (infusions of placebo to both uterine horns), EX_H – endometrial samples collected from gilts assigned to experimental group (uterine horns receiving E2 infusions), EX_C – endometrial samples collected from gilts assigned to experimental group (uterine horns receiving placebo infusion), cyclic – endometrial samples collected on day 12 of the estrous cycle, pregnant – endometrial samples collected on day 12 of pregnancy.

**Supplementary Figure 2**. Heatmap showing microarray analysis of DEGs in E2-treated endometria (833 ng of E2 /infusion), and corresponding placebo-treated endometria. Normalized expression data was clustered based on pair wise Pearson correlation using identified DEGs (red: correlation=2; blue: correlation= -2). CTRL – endometrial samples collected from gilts assigned to control groups (infusions of placebo to both uterine horns), EX_H – endometrial samples collected from gilts assigned to experimental group (uterine horns receiving E2 infusions), EX_C – endometrial samples collected from gilts assigned to experimental group (uterine horns receiving placebo infusion).

**Supplementary Figure 3.** Heatmap showing microarray analysis of DEGs in E2-treated endometria (33.3 μg of E2/infusion), and corresponding placebo-treated endometria. Normalized expression data was clustered based on pair wise Pearson correlation using identified DEGs (red: correlation=2; blue: correlation= -2). CTRL – endometrial samples collected from gilts assigned to control groups (infusions of placebo to both uterine horns), EX_H – endometrial samples collected from gilts assigned to experimental group (uterine horns receiving E2 infusions), EX_C – endometrial samples collected from gilts assigned to experimental group (uterine horns receiving placebo infusion).

**Supplementary Figure 4.** Heatmap showing microarray analysis of DEGs in endometrial samples collected on day 12 of pregnancy and day 12 of the estrous cycle. Normalized expression data was clustered based on pair wise Pearson correlation using identified DEGs (red: correlation=2; blue: correlation= -2). Cyclic – endometrial samples collected on day 12 of the estrous cycle, pregnant – endometrial samples collected on day 12 of pregnancy.

**Supplementary Figure 5.** Interaction network created for selected up-stream regulators: prostaglandin E2, progesterone, estradiol-17β, transforming growth factors beta (TGFB1-3), prolactin (PRL) and interferon gamma (IFNG) potentially regulating DEGs identified in endometrial samples on day 12 of pregnancy.

**Supplementary Figure 6.** Interaction network created for selected up-stream regulators: prostaglandin E2transforming growth factors beta 1 (TGFB1), prolactin (PRL) and interferon gamma (IFNG) potentially regulating DEGs identified in endometrial samples treated with E2 (833 ng of E2/infusion) *in vivo*.

**Supplementary Figure 7.** Interaction network created for selected up-stream regulators: tumor necrosis factor alpha (TNFA), prostaglandin E2, and transforming growth factors alpha (TGFA), potentially regulating DEGs identified in endometrial samples treated with E2 (33.3 μg of E2 /infusion) *in vivo*.
