# Supplementary figures and images for: Estradiol-17β-Induced Changes in the Porcine Endometrial Transcriptome In Vivo
Source: Int J Mol Sci. 2020 Jan 30;21(3):890. doi: 10.3390/ijms21030890 (PMC7037416; doi:10.3390/ijms21030890)

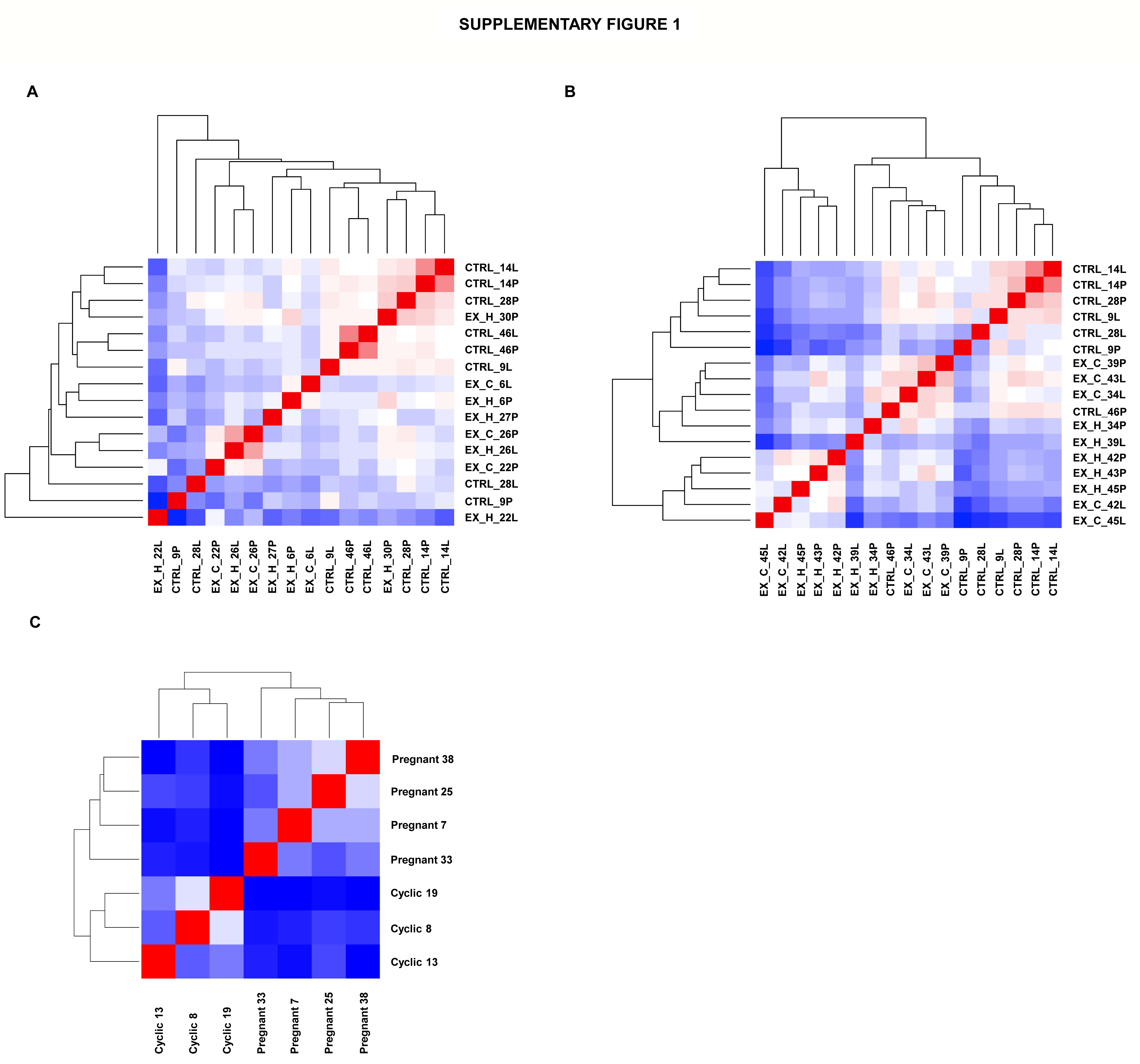

Supplement: Supplementary file 1 [file ijms-21-00890-s001.zip › Suppl Fig 1.tif]

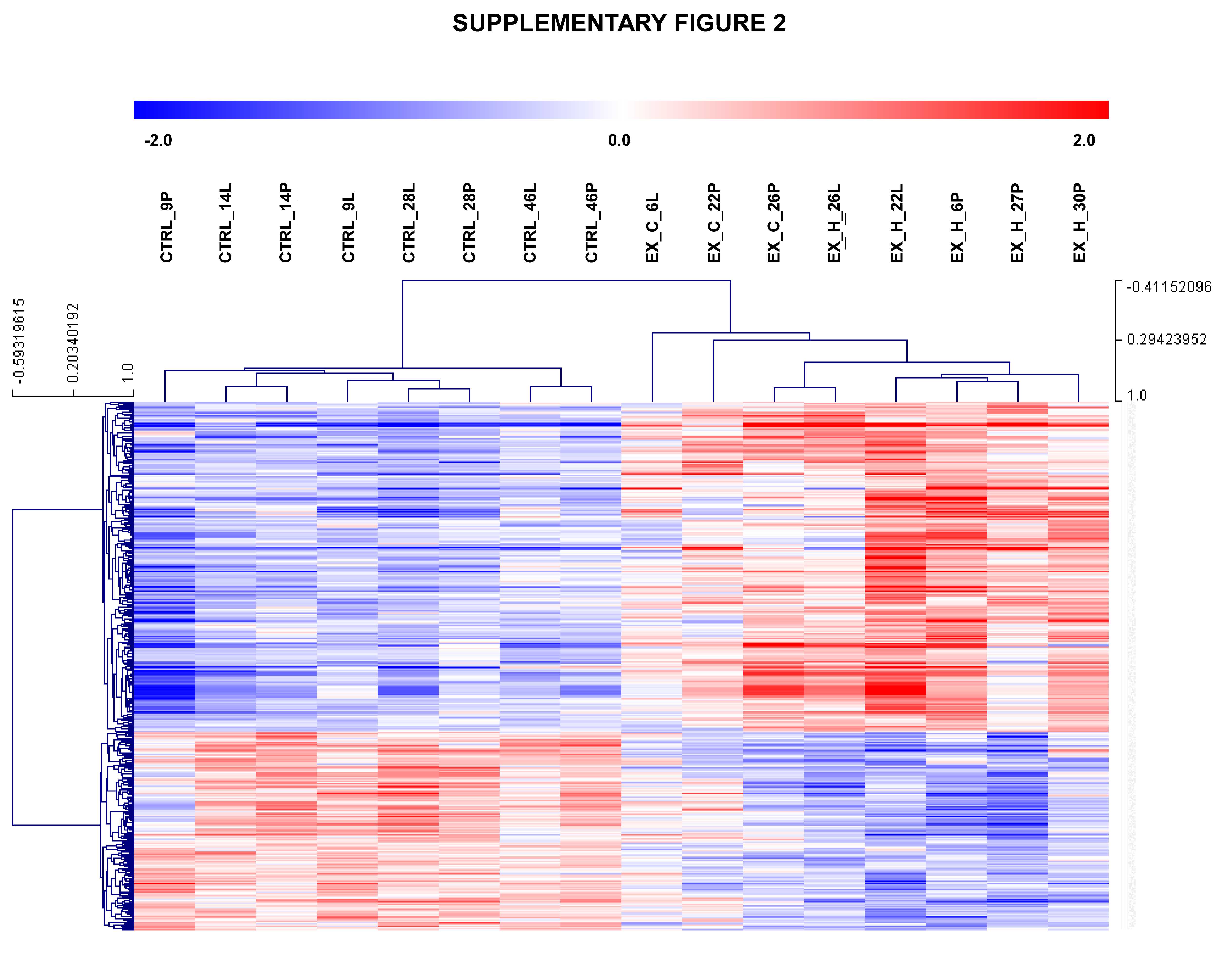

Supplement: Supplementary file 1 [file ijms-21-00890-s001.zip › Suppl Fig 2.tif]

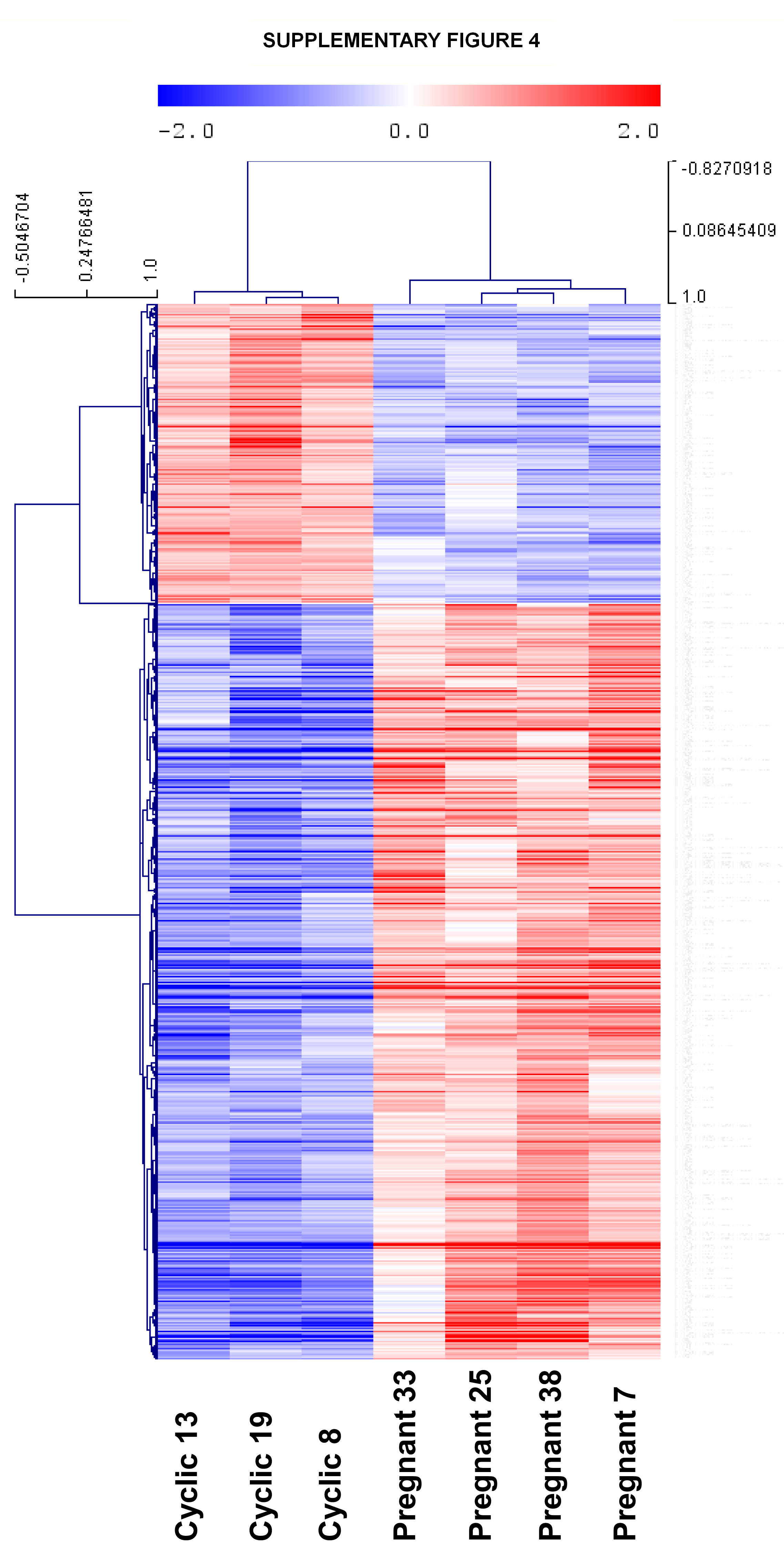

Supplement: Supplementary file 1 [file ijms-21-00890-s001.zip › Suppl Fig 4.tif]

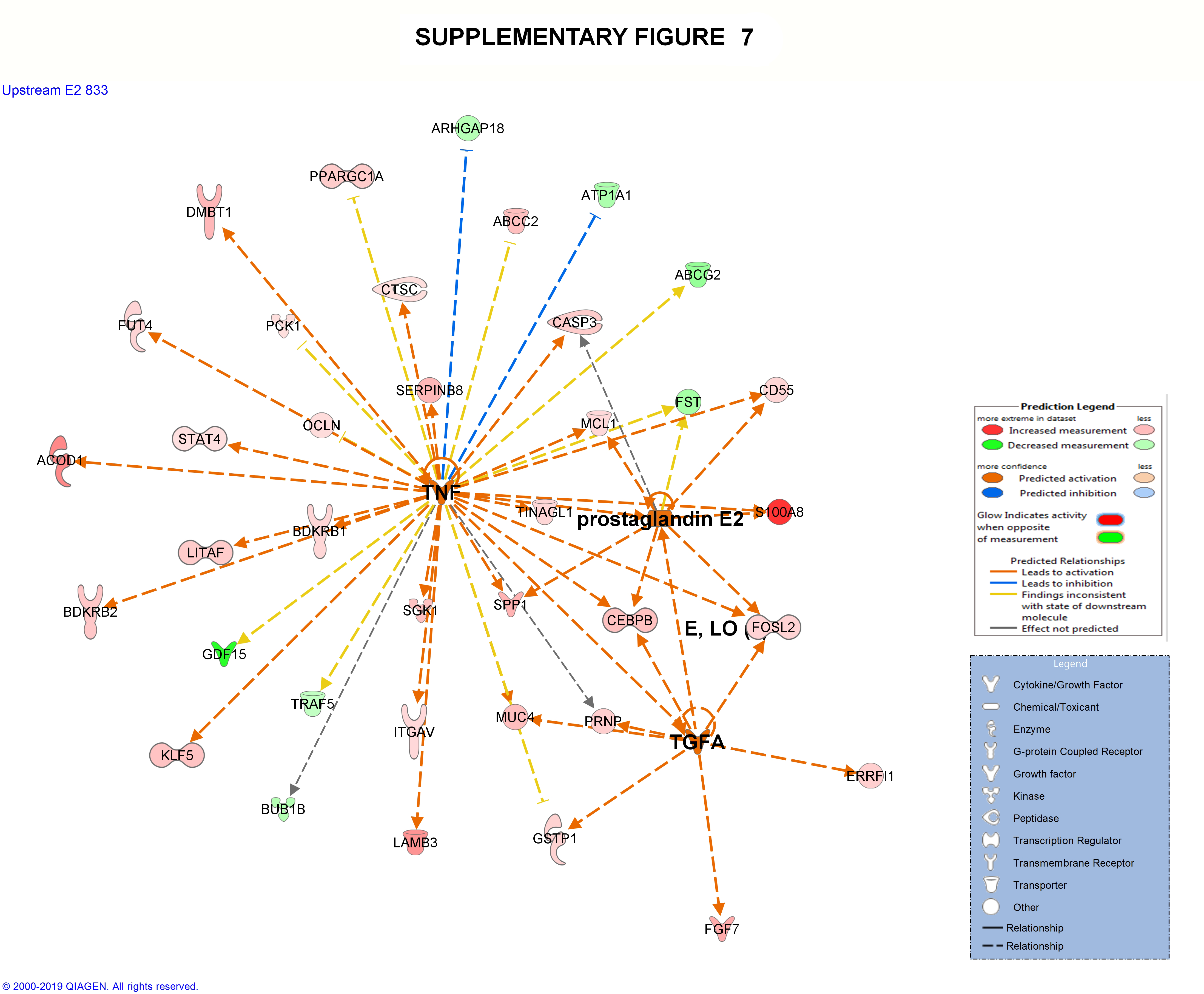

Supplement: Supplementary file 1 [file ijms-21-00890-s001.zip › Suppl Fig 7.tif]
